# The Need for Global Application of the Accountability for Reasonableness Approach to Support Sustainable Outcomes: Comment on "Expanded HTA: Enhancing Fairness and Legitimacy"

**DOI:** 10.15171/ijhpm.2016.106

**Published:** 2016-08-15

**Authors:** Jens Byskov, Stephen Oswald Maluka, Bruno Marchal, Elizabeth H. Shayo, Salome Bukachi, Joseph M. Zulu, Erik Blas, Charles Michelo, Benedict Ndawi, Anna-Karin Hurtig

**Affiliations:** ^1^Department of Public Health, University of Zambia, Lusaka, Zambia.; ^2^Faculty of Health and Medical Sciences, University of Copenhagen, København, Denmark.; ^3^Institute of Development Studies, University of Dar Es Salaam, Dar Es Salaam, Tanzania.; ^4^Department of Public Health, Institute of Tropical Medicine, Antwerpen, Belgium.; ^5^National Institute of Medical Research (NIMR), Dar Es Salaam, Tanzania.; ^6^Institute of Anthropology, Gender and African Studies, University of Nairobi, Nairobi, Kenya.; ^7^Department of Public Health, School of Medicine, University of Zambia, Lusaka, Zambia.; ^8^International Public Health Consultant, Copenhagen, Denmark.; ^9^Primary Health Care Institute (PHCI), Iringa, Tanzania.; ^10^Umeå International School of Public Health, Umeå University, Umeå, Sweden.

**Keywords:** Accountability, Health Systems, Values, Fairness, Legitimacy, Sustainability, Democratic Development

## Abstract

The accountability for reasonableness (AFR) concept has been developed and discussed for over two decades. Its interpretation has been studied in several ways partly guided by the specific settings and the researchers involved. This has again influenced the development of the concept, but not led to universal application. The potential use in health technology assessments (HTAs) has recently been identified by Daniels et al as yet another excellent justification for AFR-based process guidance that refers to both qualitative and a broader participatory input for HTA, but it has raised concerns from those who primarily support the consistency and objectivity of more quantitative and reproducible evidence. With reference to studies of AFR-based interventions and the through these repeatedly documented motivation for their consolidation, we argue that it can even be unethical not to take AFR conditions beyond their still mainly formative stage and test their application within routine health systems management for their expected support to more sustainable health improvements. The ever increasing evidence and technical expertise are necessary but at times contradictory and do not in isolation lead to optimally accountable, fair and sustainable solutions. Technical experts, politicians, managers, service providers, community members, and beneficiaries each have their own values, expertise and preferences, to be considered for necessary buy in and sustainability. Legitimacy, accountability and fairness do not come about without an inclusive and agreed process guidance that can reconcile differences of opinion and indeed differences in evidence to arrive at a by all understood, accepted, but not necessarily agreed compromise in a current context - until major premises for the decision change. AFR should be widely adopted in projects and services under close monitoring and frequent reviews.

## Background

The objective of this paper is to provide an assessment of the current status of application of the accountability for reasonableness (AFR) approach based on its described status in a 2015 publication^1^ by its key initiator Norman Daniels. That publication proposes application of AFR within an established decision-making approach for health technology assessment (HTA). Daniels publication also addresses broader considerations about AFR development and future use. It provides a current summary of AFR and finds it to be necessary as a process guidance for addressing limitations in current HTA. In brief HTA can be defined as an approach to a health outcome, technical, economic and practical feasibility assessment and comparison of technologies. These are in the publication by Daniels considered to be insufficient due to not including a specific phase of broader contextual input that is more inclusive of those who shall operate and benefit from that technology. Others have in a direct response to the publication by Daniels raised concerns associated with such consensus building as compromising overall validity of established detailed HTA procedures and refer to already existing advanced technical approaches and argue that current consultations for consistency, quality and ethics in decisions are sufficient.^2^ A massive literature exists on HTA but we consider the referenced two contemporary publications as sufficient entry points for the AFR associated issues raised in this paper.

In Daniels paper, the application of AFR to guide the HTA process is also accompanied by a discussion and some redefinition of values and expressions of value-based preferences. This is not a new discussion, but rooted in longstanding attempts to balance principles and practice for setting priorities based on advanced technical and economic rating approaches versus a more open participatory consensus building and decision-making methodologies.^3-14^

In this paper, we examine whether consensus building through AFR may be a necessary process guidance in its own right in HTA, in other already highly structured decision-making processes and in routine management.

AFR represents a process focus for agreement between individuals representing all relevant partners from all levels and not least the service users though the best possible approximation to the four conditions: Relevance, Publicity, Appeals and joint Enforcement of first three conditions.^1^ Extensive literature on AFR is referred to in the paper by Daniels. These have further developed the concept and options for application, but have not shown whether expected outcomes of fairness and legitimacy were indeed achieved and maintained. Commonly AFR has had fairly limited use for added involvement to refine rating scales for priority setting.

Only limited AFR-related implementation research has been carried out in health sector wide settings. To fill this gap, a district wide health systems AFR participatory action research was carried out in Kenya, Tanzania, and Zambia focused on developing the intervention, assessing its uptake and subsequent outcomes in selected disease or program specific areas in the three countries. This was the European Union (EU) funded (PL 517709) research project 2006-2011: Response to Accountable Priority Setting for Trust in Health Systems (REACT) for which a project overview has been published.^15^

The inclusion of stakeholders in the REACT project required explanations and interpretations of the content and wording of the original concept. The comprehensive baseline studies, the action research based application and assessment of the initial adoption of AFR by stakeholders produced a number of papers, which further clarified baseline situations and produced new knowledge on motivation and practicability of AFR.^16-22^

We laid a solid ground through assessing the understanding of values in the communities based on comprehensive qualitative approaches. Considering cultural and language differences it was clear that non-technical interpretations of versions of values such as fairness, legitimacy, responsibility, compassion, transparency, equity, and quality were quite similar in study sites in Kenya, Tanzania, and Zambia. There is of course a danger that values expressed to us represent an ideal,^23^ but even so such comparable awareness of ideals may be one stepping stone towards their realization.

The implementation did result in changes in attitudes to participation, in local structural changes for that, and in some increased service output coordination. These are important indicators for change in fairness and legitimacy and increase the likelihood for a desired change in the defined core study outcome indicators for quality, equity and trust. However, we had to realize that main study indicators remained as relevant targets for change, which were not possible to document within the project time left after the formative and baseline stages. This new insight led to important mutual learning, which has also been captured in further REACT-based publications including its final overarching project results assessment.^24^ Since project end the involved Primary Health Care Institute in Iringa, Tanzania further developed and applied REACT-based training packages^25^ and the for the local context further adapted REACT guide for AFR in 4 districts in their region. The uptake was good and local variations showed that it was considered as a highly welcomed District Health Team internal communication and management aid for a more consistent and continuous de-central priority setting and for other decision-making as well. Due to the general lack of support for such in the already by technical criteria overloaded supervisory structures and the cessation of specific funding preliminary results were reported,^26^ but further follow up was not done.

## Discussion

The paper by Daniels et al^1^ represents a clear and highly relevant AFR guidance for HTA, but the actual action to scale up AFR through managers of health systems still seems evasive.

We think that it is important to bring the now available insights into the debate on the potential and a recommended further application of AFR.

The so far promising experiences from AFR application need to be further tested in a more equal balance and mutual acceptance between professional and technical priority setting versus participatory preferences and values. The concerns that are raised by those who primarily support the consistency and objectivity of selected evidence must be considered, but the inherent differences and different justification for the two sides must be respected and balanced. One cannot do without the other. We should not waste time on the fruitless discussions on not comparable premises concerning which is more important, but just coordinate and balance them in their own right. Illustrative comparable dilemmas at different levels are the never ending discussions of relative importance of quantitative and qualitative research paradigms, and of ethical and economic priorities for availability of the latest treatment for individuals versus addressing the main conditions for improving population health.

The debate on values and their use has continued, but not been concluded into agreed operational approaches. The current paper by Daniels et al^1^ and other papers^5,6,8,13^ are not consistent on their definitions of values, so that leaves uncertainty and lack of buy in from practitioners. A previous consensus on overriding global values (primary healthcare – PHC - as defined in Alma Ata 1978)^27^ included the guiding principles of equity, community participation, appropriate technology, focus on prevention and inter sectorial collaboration. They created new global dynamics and processes, which were later fragmented into more separately guided and funded programmatic primary contact level interventions often bypassing the still weak national health systems and thus delaying country-based systems capacity development. That situation still prevails. These are among principles and values, which can be considered in an AFR process. Other commonly stated values are equity, efficiency, and quality. Within a fixed resource frame these stand in competition and an assessment of their respective influence on strategies and priorities must be made explicit.

The publication by Daniels et al strongly supports participation of stakeholders in both the formative and assessment stages, but the process for achieving such is not included. Developing and testing incorporation of AFR in existing strategic, annual, and other monitoring cycles will be an essential first step if AFR is to assist in guiding the processes towards better solutions. It is not enough to just add an AFR component to selected highly pre-structured and difficult to influence areas such as HTA.

The role and expertise of participants must be differently conceptualized. Technical experts and higher level managers are well-versed with technical approaches. Beneficiaries, users, and communities relate to a range of political, social, and cultural values. They are experts on their own broad and often changing range of values and preferences. The need for their influence on decisions is comprehensively covered in the more general literature also outside the health sector.^28-32^ We are not venturing further into that literature, but find AFR arguments well-defined within that frame.

By its process focus AFR in its simplest face value of the conditions and the process orientation bypasses the necessity for full detailed insight and sharing of all factors to guide preferences. AFR facilitates joint ownership, commitment and responsibility for actual decisions as fully agreed or accepted as necessary compromises under the circumstances. There will of course be different needs of involvement, orientation and time depending on the type of issue and the level of decision-making, but the operational and community level decisions must be respected by the higher levels and be responded to in mutually understood and acceptable terms. That raises the question whether it can be termed unethical not to actively accommodate AFR guidance to ensure collaboration for better health.

The publication by Daniels et al also points out vested interests as constraining factors for adequate priority setting. This is a particular danger for donor or commercial consultant driven assessments and plans which tend to simplify reasoning and neglect context and therefore need in country checks and balances. AFR may provide some of that. Additional vetting of methods quality may also be commissioned to thus defined branches and staff of Public Health research and training institutes. They and their universities are bound by quality criteria as justification for their existence.

The way ahead for AFR can be a continuous and in county approved and supervised monitoring of progress in hopefully decreasing gaps in the compliance with the conditions. If agreed that the four conditions are most likely to promote better decisions through participatory principles, then little further justification is necessary before launching a scaled up practice even up to national level and maybe even beyond the health sector.

Finally reasonableness is by many seen as difficult to understand and translate. To get a wider public uptake of AFR, it will “sell” better if AFR is explained as Accountability for Fairness and Rights (rights illustrating the legitimacy aspect).

## Conclusion

AFR provides a means for better and more sustainable choices on health for all and for everyone – in line with the Sustainable Development Goals overriding statement “leave no one behind.” It does not contradict or constrain accountable health sector organizational and technical development but assists in sharing and coordinating interests and agendas, for which a legitimacy assessment is needed. AFR is, thus, ready for universal application combined with close monitoring, frequent reviews and research.

## Ethical issues

Not applicable.

## Competing interests

Authors declare that they have no competing interests.

## Authors’ contributions

JB conceptualized, initiated and coordinated work for this publication and drafted the paper, SOM represented the Tanzanian data set, BM ensured consistent scientific quality of underlying data across the three study countries, EHS provided additional data from Tanzania, SB represented the Kenyan data set, JMZ represented the Zambian data set, EB represented major international insight, CM supported the drafting in the Department of Public Health in Lusaka, Zambia, BN coordinated AFR training and contributed to the AFR guide, AKH provided conceptual backing to the drafting. All except EB were participants and among authors to the earlier joint AFR intervention and study publications. All read and approved the submission.

## Authors’ affiliations

^1^Department of Public Health, University of Zambia, Lusaka, Zambia. ^2^Faculty of Health and Medical Sciences, University of Copenhagen, København, Denmark. ^3^Institute of Development Studies, University of Dar Es Salaam, Dar Es Salaam, Tanzania. ^4^Department of Public Health, Institute of Tropical Medicine, Antwerpen, Belgium. ^5^National Institute of Medical Research (NIMR), Dar Es Salaam, Tanzania. ^6^Institute of Anthropology, Gender and African Studies, University of Nairobi, Nairobi, Kenya. ^7^Department of Public Health, School of Medicine, University of Zambia, Lusaka, Zambia. ^8^International Public Health Consultant, Copenhagen, Denmark. ^9^Primary Health Care Institute (PHCI), Iringa, Tanzania. ^10^Umeå International School of Public Health, Umeå University, Umeå, Sweden.
